# Assessment of school health services in primary schools in Gwagwalada area council, Federal Capital Territory, Nigeria

**DOI:** 10.11604/pamj.2022.41.251.27372

**Published:** 2022-03-28

**Authors:** Usman Abiola Sanni, Kareem Iwunmole Airede, Emmanuel Ademola Anigilaje, Uduak Mayen Offiong

**Affiliations:** 1 Department of Paediatrics, Federal Medical Centre, Birnin Kebbi, Kebbi State, Nigeria,; 2 Department of Paediatrics, University of Abuja, Abuja, Nigeria,; 3 Department of Paediatrics, University of Abuja Teaching Hospital, Abuja, Nigeria

**Keywords:** School health services, public, private, primary schools, Gwagwalada area council, Federal Capital Territory, Nigeria

## Abstract

**Introduction:**

School Health Service (SHS) is one of the five main components of the School Health Programme aimed at ensuring every child remains healthy to benefit maximally from his/her education. This study aimed to assess the level of implementation of SHS in primary schools in the Gwagwalada Area Council of the Nigerian Federal Capital Territory.

**Methods:**

a cross-sectional study was carried out in primary schools in Gwagwalada Area Council using a weighted School Health Service assessment checklist.

**Results:**

a total of 146 primary schools were studied. Ninety-five (65.1%) of the schools had no health personnel. First aid was offered by 129 (88.4%) of the schools for medical emergencies. All schools sent children with communicable diseases home. None of the public schools assessed had an ambulance or a sickbay. A total of 44 (30.1%) schools, made up of 8 (20.0%) public and 36 (34.0%) private schools, attained the acceptable minimum score of 19. The mean scores on school health services by the private and public schools were 16.31±3.96 SD and 16.23±2.87 SD respectively out of the attainable maximum score of 45 (t=0.145, p=0.885).

**Conclusion:**

the level of implementation of SHS in Gwagwalada Area Council is inadequate though with a slightly better situation in the private schools. For more effective SHSs in the study area, there is the need for the provision of sufficient human resources and facilities by stakeholders in Gwagwalada Area Council primary schools.

## Introduction

World over, there has been a significant increase in school enrolment over the past decade [1, 2]. In many homes across the world, children get enrolled in pre-primary schools (daycare and crèche) as early as five to six months of life as mothers need to return to work [3]. Furthermore, the number of children attaining school age has increased; estimated to be about 18% and 25% of the world´s and Nigeria´s populations respectively and this figure is rising [3-5]. Even though parents are in the best position to note any health problem with the child, most parents have little time for their children, spending most of their time at work or commuting to and from work, leaving their children in the care of schools for longer hours each day [6]. This results in the abdication of the parental role of care-giving to the schools. Having effective school-based health services, therefore, cannot be overemphasized.

School health service is one of the five components of the school health programme aimed at ensuring every child remains healthy to benefit maximally from his/her education [7]. Its proper implementation will also help in the attainment of Sustainable Development Goals (SDG), that are related to education and health [8]. In a national study conducted in 2003 by the Nigerian Federal Ministry of education in collaboration with the World Health Organization (WHO), the quality of healthcare services in schools across Nigeria was found to be suboptimal [7]. Various other studies in Nigeria, mainly from the southern regions, also showed an unsatisfactory implementation of school health services/programme [9-14]. To improve school health services implementation in Nigeria, there is a need to obtain data from every region of the country. At present, there are limited studies on school health services in Northern Nigeria [11, 14], including the Federal Capital Territory (FCT).

Getting data from Northern Nigeria, where primary school attendance is abysmally below the national average, is particularly important since the availability of adequate SHSs had been shown to increase school enrolment [14, 15]. Furthermore, even though policies on school health programmes were developed in the FCT since 2006 [7, 16], there have been no published studies on the assessment of the implementation of SHSs in primary schools in the FCT in general, and Gwagwalada Area Council (GAC) in particular.

The objective of this study was to assess the status of the SHS in primary schools in Gwagwalada Area Council of the Nigerian Federal Capital. This would, in addition to assessing the situation of the SHSs in the capital of Nigeria, add to the data on SHSs of the Northern Nigeria. It would also serve as a reference point for subsequent review.

## Methods

**Study location:** the study location was Gwagwalada Area Council (GAC). It is one of the six Area Councils of the Nigerian Federal Capital Territory, located in the North Central region. Its 2016 projected population was 402 000 people, consisting of the indigenous Gbagyis and Bassa with other settlers such as the Hausas, Fulanis, Koros, Yorubas and Ibos [17, 18]. The study was carried out over seven months from April to October 2017. The total number of registered primary schools in the Area Council was 291 consisting of 80 public and 211 private schools. The authority of the primary schools lies with the Universal Basic Education (UBE) Board and Zonal Education Office (ZEO), Gwagwalada, Federal Capital Territory.

**Study design:** the study was a descriptive cross-sectional type.

**Study population:** public and private primary schools.

**Sample size determination:** the sampling frame was 291, consisting of 80 public and 211 private primary schools. A sampling ratio of 50% of the 291 schools was used, which allowed for a precision of 5% at a 95% confidence interval [11, 19]. This gave a sample size of 146 schools.

**Sampling technique:** a stratified random sampling method was used to stratify the primary schools into public and private schools. The sampling ratio of 50% applied for sampling in each school category resulted in the selection of 40 public and 106 private schools respectively, giving a total of 146 schools for the study. The 146 schools were randomly selected from the list of schools by balloting. Only registered primary schools located within GAC whose head teachers consented to the study were recruited for the study.

**Ethical consideration:** the ethical approval for the study was obtained from the University of Abuja Teaching Hospital´s Research and Ethics Committee (FCT/UATH/HREC/PR/034). Approvals were also obtained from Universal Basic Education Board and Zonal Education Office, GAC. Consent was obtained from each participant. Confidentiality was assured by the use of codes on the assessment forms.

**Data collection:** school Health Services Evaluation checklist was the study instrument. The checklist was adapted from “School Health Practice” by Anderson and Creswell [20]. It has sections on the school administration data, and various components of SHSs such as health personnel in the school, health facilities, care of emergency illness, control of communicable diseases, record keeping, nutritional services (Annex 1). The evaluation checklist was completed for each school by direct interview/inspection and report by the head-teachers where observations did not apply. The acceptable maximum and minimum attainable scores by each school, to be classified as having effective SHS, were 45 and 19 respectively Health (or class teachers), health staff and two pupils were also interviewed separately to ensure accuracy. The pupils were randomly selected from the primary six classes. The respondents were interviewed separately. A pilot study was carried out in two (one public and one private) schools in Kwali Area Council (a neighbouring Area Council) to identify problems that could be encountered in the administration of the questionnaire. No modification was, however, required. The findings from the pilot study were not included in the data collected.

**Data analysis:** the data obtained were sorted according to school ownership into private/public. Analysis was done using Statistical Programme for Social Science (SPSS) version 20. Categorical variables were reported as proportions, while continuous variables as means and standard deviations. Pearson chi-square test or Fischer´s exact test (where appropriate) was used to compare frequencies in the contingent tables, as well as differences between proportions. Student t-test, however, was used to compare group means. P-values of less than 0.05 were regarded as significant in all statistical tests of significance.

## Results

**Demographics of the schools:** one hundred and forty-six (146) primary schools consisting of 40 public and 106 private schools were studied. There were a total of 52 756 pupils, consisting of 26 774 females and 25 982 males in the schools surveyed. Public schools had more pupils, 38 685 (73%) compared to 14 071 (27%) in private schools. Also, there were a total of 2 154 teachers in the schools. As of the time of the study, mean school-age was 18.98 (±9.67 SD) years. Public primary schools in the Area Council had a mean age of 27.60 ± 10.26 years, while that of private schools was 9.74 ± 3.04 years (t= 0.689, p<0.001). There was a significant difference between public and private schools (37.5% versus 14.2%, respectively) for the presence of school health committee (p=0.002).

**Health personnel, health appraisal, and care of emergency illness in the schools:** Table 1 shows the distribution of health personnel, health assessment and care of emergency illness in the schools surveyed. Of the health personnel in the schools surveyed, 43 (29.5%) were trained First Aiders. The majority (65.1%) of the schools had no health personnel. As regards health appraisal in the schools, 143 (99.3%) schools {40 (100.0%) public and 105 (99.3%) private} carried out routine inspection of pupils´ teeth, finger nails and school uniform. Significantly more private {62 (58.5%)} than public {5 (15.0%)} schools requested for pre-entry medical screening test from the pupils; χ^2^=22.075, p value <0.001. In the event of emergency illnesses in the schools, 38 (95%) public and 91 (85.8%) private schools administered first aid treatment (p=0.124). While 143 (97.9%) schools would notify the parents of any emergency illness of their children immediately, 126 (86.3%) schools would convey such children home after treatment.

**Table 1:** health personnel, health assessment/care and care of emergency illness in schools

Health personnel	Public N=40 n(%)	Private N=106 n(%)	Total N=146 n(%)	χ^2^	P-value
Trained first aider	12(30.0)	31(29.2)	43(29.5)	0.008	0.929
Nurse/midwife	0(0.0)	5(4.7)	5(3.4)	2.361#	0.124
Doctor Ѫ	0(0.0)	3(2.8)	3(2.1)	1.156#	0.282
Nil	28(70.0)	67(63.2)	95(65.1)	0.598	0.443
**Health appraisal ψ**					
Routine inspection by teachers	40(100.0)	105(99.1)	145(99.3)	0.380	0.538
Referral services	36(90.0)	88(81.1)	124(84.9)	1.106	0.293
Pre-entry screening test	6(15.0)	62(58.5)	68(46.6)	22.075	<0.001*
Supervision of health of physically challenged	7(17.5)	18(17.0)	25(17.1)	0.006	0.941
Periodic medical examination	3(7.5)	16(15.1)	19(13.0)	1.480#	0.224
**Care of emergency illness ψ**					
First Aid treatment	38(95.0)	91(85.8)	129(88.4)	2.364	0.124
Treatment given recorded	16(40.0)	38(35.8)	54(37.0)	0.215	0.643
Notification of parent immediately	38(95.0)	105(99.1)	143(97.9)	2.375	0.123
Transport child to the nearest health post	38(95.0)	97(91.5)	135(92.5)	0.508	0.476
Convey child home after treatment	37(92.5)	89(84.0)	126(86.3)	1.791	0.181

Key; #- Fischer's exact test *: p-value <0.05 (i.e. statistically significant) χ^2^: Chi-square Ѫ: Occasional consult on request ψ: multiple responses possible

**Treatment facilities and content of first aid boxes in the schools:** treatment facilities within the schools and the contents of the First Aid boxes are shown in Table 2. First Aid boxes were present in 39 (97.5%) public and 90 (84.9%) private schools, with a significant difference between the 2 school categories, χ^2^=4.477, p=0.034. A sickbay was present in 24 (22.6%) private schools and none of the public schools. Concerning the content of the First Aid boxes, a total of 114 (78.1%) schools had cotton wool in their First Aid boxes. Analgesics were present in 70 (47.9%) schools {19 (47.5%) public and 51 (48.1%) private} with no significant difference between the two school categories (p= 0.947). None of the schools had materials for wound suturing. Also, none of the schools had emergency drugs like diazepam, glucose, or relief medications for asthma.

**Table 2:** treatment facilities and content of the first aid boxes

Treatment facilities ψ	Public N=40 n (%)	Private N=106 n (%)	Total N=146 n (%)	χ^2^	P-value
First aid box	39(97.5)	90(84.9)	129(88.4)	4.477	0.034*
Health room/dispensary	0(0.0)	24(22.6)	24(16.4)	10.838#	0.001*
School bus/ambulance	0(0.0)	45(42.5)	45(30.8)	24.547#	<0.001*
School telephone services Ф	38(95.0)	105(99.1)	143(97.9)	2.3755	0.123
**Content of the first aid boxes ψ**					
Analgesics	19(47.5)	51(48.1)	70(47.9)	0.004	0.947
Anti-malarial	1(2.5)	10(9.4)	11(7.5)	2.004#	0.157
ORS	3(7.5)	6(5.7)	9(6.2)	0.170#	0.680
Disinfectant	35(87.5)	75(70.8)	110(75.3)	4.383	0.036*
Cotton wool	33(82.5)	81(76.4)	114(78.1)	0.628	0.428
Plaster	33(82.5)	72(67.9)	105(71.9)	3.055	0.080
Bandages	34(85.0)	73(68.9)	107(73.3)	3.861	0.049*
Empty first aid boxes	2(5.0)	6(5.7)	8(5.5)	0.024#	0.876

* : p value< 0.05 (i.e. statistically significant) #: Fischer's exact test χ^2^: Chi square ψ: multiple responses possible Ф: owned by school management for information dissemination including health-related especially in emergency situations

**Control of communicable diseases, nutritional services and medical record-keeping in schools:** control of communicable diseases, nutritional services and medical record-keeping in the schools are illustrated in Table 3. All schools sent children with communicable diseases home. Only one private school made an exemption to this during examinations. There were only two schools which routinely carried out immunization of their school populations during outbreaks of diseases. School meals were available in 33 (22.6%) schools. In the 21 (45.0%) public and 12 (8.3%) private schools where this service was available, commercial food vendors were the only sources of the school meals. None of the schools served free meals. Eight (20.0%) of the 21 public schools mandated medical screening of the food vendors. While 94 (64.4%) schools had no medical record, such records in 5 (3.4%) schools {1 (2.5%) public and 4 (3.8%) private} were cumulative, though not transferrable.

**Table 3:** control of communicable diseases/care and care of emergency illness in schools

Control of communicable diseases ψ	Public N=40 n(%)	Private N=106 n(%)	Total N=146 n(%)	χ^2^	P-value
Send child home	40(100.0)	106(100.0)	146(100.0)	-	-
Health talks ¥	33(82.5)	87(82.1)	120(82.2)	0.004	0.952
Isolation in a health room	0(0.0)	1(0.9)	1(0.7)	0.380#	0.538
Immunization	2(5.0)	2(1.9)	4(2.7)	0.380#	0.538
**Nutritional services ψ**					
Nutrition demonstration class	22(55.0)	59(55.7)	81(55.5)	0.005	0.943
School farm	18(45.0)	24(22.6)	42(28.8)	7.085	0.008*
School meals	18(45.0)	15(14.2)	33(22.6)	15.789	<0.001*
Nil	2(5.0)	30(28.3)	32(21.9)	9.214#	0.002*
Medical record keeping					
Nil	25(62.5)	69(65.1)	94(64.4)	0.085	0.770
Available, non-cumulative	14(35.0)	33(31.1)	47(32.2)	0.199	0.655
Cumulative, non- transferrable	1(2.5)	4(3.8)	5(3.4)	0.142*	0.706
Cumulative and transferable	0(0.0)	0(0.0)	0(0.0)	-	-

* -p value< 0.05 (i.e. statistically significant), #: Fischer´s Exact test, ψ: multiple responses possible, ¥: usually by staff, but occasionally undertaken by guest

**The proportion of schools with adequate SHS as well as SHS Scores and their mean comparison:** overall, forty-four (30.1%) schools made up of 8 (20.0%) public and 36 (34.0%) private schools attained the acceptable minimum score of 19 in school health services. The mean scores on school health services by the private schools was 16.31±3.96 SD as compared to the public schools with a mean of 16.23±2.87 SD out of the maximum attainable score of 45. An independent t-test for equality of these means showed no statistically significant difference (t=0.145, p=0.885).

## Discussion

Overall, the level of implementation of school health services (SHSs) in primary schools in Gwagwalada Area Council, Federal Capital Territory was poor with only a few schools attaining the acceptable minimum score despite being located in the country´s Federal Capital Territory. The findings of this study were also similar to studies from other parts of Nigeria and other African countries [10, 12, 13, 21-23]. This implies that the school-based health services may not be adequate to meet the health needs of pupils in most of the schools.

Private schools performed somewhat better than public schools in the implementation of SHSs, similar to studies in other parts of Nigeria and other developing countries [11, 12, 24-27]. This may not be unconnected with the profit-making orientation of proprietors of such private schools making them more committed to providing necessary facilities. Hence, they tend to offer quality services, including school health activities to attract parents. This may not be the same in public schools where enrolment was not a problem due, perhaps, to the low or non-existence of fees, thus attracting a large number of children. Also, it could be because most of the private schools were relatively new compared to the public ones. This implies, perhaps, that their structures and facilities were newer, contributing to their relatively better score. In addition, it could be that public schools were poorly funded by the government; thereby hindering the acquisition and maintenance of structures that would help the school health service.

In the current study, only a few schools had any form of health personnel; mainly trained first aiders. Only private schools benefitted from the services of nurses and doctors. This may be due to the higher school fees charged by these private schools which made it possible for some proprietors to afford the services of these health workers. In this study, the 4.7% of the schools with nurses was at variance with 31.7% of schools reported by Kuponiyi *et al*. [26] in Ogun state primary schools. The observed difference may be location dependent; Ogun state is located in South-Western Nigeria with more health personnel. These findings, however, contrasted the finding in the United States of America where more than 82% of the country´s public schools employed school nurses; 63% of which employed full-time school nurses [28]. The observed difference may be a reflection of differences in resource availability between developed and developing countries. Health personnel have a central and coordinating role in SHSs [9]. The absence of such personnel in schools may affect other aspects of the school health services.

Treatment facilities available in this study for the care of onsite illnesses included first aid boxes in both public and private schools. The fact that most (88.4%) of the schools had first aid boxes, though less than 94.6% reported by Adeniran *et al*. [29] study in Lagos, with only 5.5% being empty is commendable. This was better than 66.7% reported in an Indian study [27]. The presence of first-aid boxes in most schools in GAC was, perhaps, a reflection of their importance as perceived by the school authorities. The main contents of the first aid boxes in this study were the wound dressing materials and analgesics though none of the boxes had materials for suturing wounds. The finding in this survey was similar to those of Osuorah *et al*. [24], Qureshi *et al*. [30] and Ofovwe *et al*. [31]. The stocking of first aid boxes with wound dressing materials and analgesics by most schools may be premised on the reason that children sustain a varying degree of injuries in schools due to their adventurous nature and high physical activity, thereby requiring dressing materials to prevent wound infections and analgesics to relieve pain.

No public school had a sick bay nor a school bus/ambulance to convey sick children home or to the hospital. This could be a reflection of the status of school health services in these public schools. The finding of 16.4% of schools, all privately owned, in this study having a clinic/ sickbay was better than 0% reported by Qureshi *et al*. [30] in Karachi, Pakistan. The presence of health personnel in a higher proportion of schools in the Nnewi study (82.1% versus 34.9% in GAC), however, may be a reason for the presence of sickbay in a higher proportion (69.9%) of schools surveyed in Nnewi LGA, Nigeria (since schools would have to provide sickbay for the health personnel to treat sick pupils) [24]. This might be an illustration of the significance of having health personnel in schools.

Availability of school buses in a third of the schools, all privately owned, was higher than that reported by Kuponiyi *et al*. [26] in their study of primary schools in Ogun State, Nigeria. The absence of a school-owned mode of transportation in the public schools in the current study was similar to the finding of Asiabaka in Imo state [32]. The presence of a school bus would facilitate the transportation of sick children to hospitals. Despite the challenges of few health personnel and treatment facilities, most schools reported instituting first aid before transporting children to health posts for further treatment in the event of emergency illnesses/injuries.

The commonest health appraisal undertaken by most of the schools surveyed was routine early morning inspection of the pupils. This was conducted two or three times per week in most of the schools. Such high participation in this activity was similar to finding in other Nigerian studies [26, 33, 34]. This, unlike caring for the sick children, requires no training or skills and without much effort teachers were able to carry out the activity; perhaps, the reason for its high rate in these studies. This would encourage inculcation of good personal hygiene, in addition to early detection of apparent illnesses (like skin diseases), in school children. Pre-entry medical screening, on the other hand, was requested by less than half (46.6%) of the schools, mostly private schools, similar to 45% recorded by Ofovwe and Ofili [31]. None of the schools made it a compulsory admission prerequisite. Flexibility on the screening policy by public schools could have been premised on the government policy of allowing all Nigerian children access to Universal Basic Education (UBE).

As regards supervision of children with special health needs and the physically challenged, this appraisal was inadequately carried out in the study area, as only 17.1% of surveyed schools practiced this; similar to the finding in rural Zimbabwe [35]. These were, however, contrary to the situation in Spain, wherein a region, up to 70% of schools were actively involved in the health needs of deaf pupils [36]; thereby making schools fulfil the quality of being a health-promoting setting. To control communicable diseases, all the schools sent sick children home similar to the practice in other parts of the country [12, 13, 25]. The fear of contracting such communicable diseases by other members of the school population possibly informed this decision in almost all the schools. Other possible reasons for this action could be the non-availability of isolation rooms and/or health personnel and essential drugs to handle such conditions in the schools.

Another aspect of control of communicable diseases evaluated in this study was the administration of immunizations. Even though school-aged children are not the primary target of routine immunization schedules in most countries, the availability of immunization services in schools could cover the gap in vaccination reported by the WHO [37], as well as serve for the administration of boosters for routine vaccination and during epidemics. This service was absent in the vast majority of the schools surveyed, similar to findings from other Nigerian studies. [12, 25] This was in contrast to results from high-income countries where most school-aged children receive vaccinations in schools [38]. Non-adoption of the WHO recommendation of school-based immunization for delivery of booster for routine vaccination in Nigeria may be an explanation for the observed difference. The finding in this study further buttressed a systematic review by UNICEF that school-based immunization was not commonly used in low and lower-middle-income countries [38]. The implication of non-availability of immunization services in primary schools in the study area is that any child who had not been fully immunized for one reason or the other would still not get those missed vaccines despite attending school; thereby missing the opportunity of vaccination which had been described as the most effective public health measure for infectious disease control, second only to the provision of clean drinking water [39].

Most of the schools surveyed kept no health record; where kept, the records were neither detailed nor transferrable. This was in contrast to the practice of schools in Belgaum, India where all schools kept health records [27]. Perhaps, most of the schools studied were unaware of the importance of proper record keeping and this may be the reason for this abysmal finding. It could also be a reflection of the inadequate health personnel (who would have ensured proper records were kept) in the schools. This would make monitoring and evaluation of the school health service difficult in the study area.

Nutritional services were poor in the study area. School meals were available in less than a quarter (22.6%) of schools in GAC compared to Ilesha East LGA, Osun State [12], where all schools provided school meal. The difference in the two studies could be accounted for by the Federal Government of Nigeria school feeding programme that was ongoing in Osun State including Ilesha East LGA at the time of the study that provided free school meals to the pupils. There was no similar activity in place in GAC (and Abuja as a whole) at the time of this study. Where school meals were served, it was on fee-service bases. The absence of a free school-feeding programme could have a negative impact on the nutritional status of the school children in the study area.

It is part of school health that screening of school food vendors is carried out. This crucial public health function though performed in some of the public schools, the numbers were still very poor. This finding was in contrast with the practice of schools in Ghana where food vendors were only permitted to sell mid-day meals to students after undergoing series of tests with certification yearly, in addition to the use of apron and head covers as reported by Monney *et al*. [40] in Konongo. This observed difference might be due to the poor oversight function of the Zonal Education Office or other Agencies saddled with such responsibility. The corollary to this is that majority of pupils in the study location may be exposed to potential public health hazards through consumption of food from unscreened food vendors.

The availability of a school farm and nutrition demonstration classes can also promote improved nutrition. The proportion (more than half) of the schools that carried out nutrition demonstration classes in the current survey was higher than those reported in some Southern Nigerian studies [13, 25]. School farming, on the other hand, was practised in 28.8% of the schools surveyed, compared to 67% noted by Ezeonu *et al*. [21] in Abakaliki. The higher proportion noted in Abakaliki may be related to the widely practised farming activity in their environment. The farm produces from these school farms could be used in nutrition demonstration classes as well as in the provision of school meals thereby improving the nutritional status of the school children.

## Conclusion

The status of implementation of school health service in Gwagwalada Area Council is poor reflecting a deficit in both material and human resources necessary for its implementation. There is a need, therefore, for the provision of school health facilities in Gwagwalada Area Council primary schools, including first aid boxes (stocked with essential drugs), and sickbays. Each school should recruit health personnel or train at least one staff in first aid. The school feeding programme should also be extended to primary schools in Gwagwalada Area Council.

### What is known about this topic

- School health service implementation in most Southern Nigerian states is poor;
- School health service implementation is better in private than public schools in most Nigerian studies;
- There is a paucity of data on the assessment of school health service implementation in Northern Nigeria.

### What this study adds

- Implementation of school health services in primary schools in Gwagwalada Area Council (GAC) of the Nigerian Federal Capital Territory, where the Nigerian school health policy and implementation guidelines were developed, is poor;
- Performances of the public and private primary schools in GAC were comparable though with slightly better situation in the private schools;
- Nutrition demonstration classes are an integral part of nutritional services in quite a number of primary schools in GAC. Screening of food vendors, on the other hand, is mainly undertaken by the public schools.

## Annexe

**Annex 1**: school health services evaluation scale

## References

[1] UNICEF Annual Report 2015.

[2] Amanchukwu RN, Ololube NP (2015). Excellent school records behaviour for effective management of educational systems. Hum Resour Manag Res.

[3] Oguji CN, Ezedum CE (2003). School health services. School health education.

[4] Federal Ministry of Health Nigeria The National Primary Health Care Development Agency: Comprehensive EPI multi-year plan 2011-2015. Abuja.

[5] Akani NA (2016). The Advocate?: For richer for poorer in sickness and in health, preserving the heritage. Inaugural Lecture Series (University of Port Harcourt, Nigeria).

[6] Morokola AO School health programme 2003 Ibadan.

[7] Federal Ministry of Education, Nigeria (2006). National school health policy Federal Ministry of Education. Abuja.

[8] United Nations Sustainable Development Goals platform Sustainable Development Goals.

[9] Akani NA, Nkanginieme KEO, Oruamabo RS (2001). The school health programme: A situational revisit. Niger J Paediatr.

[10] Ademokun MO, Osungade KO, Obembe TA (2014). A qualitative study on the status of implementation of school health programme in South Western Nigeria: Implications for healthy living of school-age children in developing countries. Am J Educ Res.

[11] Toma BO, Oyebode T, Toma GIO, Agaba E (2014). School health services in primary schools in Jos. Open Sci J Clin Med.

[12] Olatunya O, Oseni S, Olaleye A, Akani NA, Oyelami O (2015). School health services in Nigeria: A sleeping giant?. Afr J Health Sci.

[13] Oyinade OO, Ogunkunle OO Olanrewaju DM (2014). An evaluation of school health services in Sagamu, Nigeria. Niger J Clin Pract.

[14] Dania O, Adebayo AM (2019). School health program in Nigeria?: A review of its implementation for policy improvement. American Journal of Educational Research.

[15] USAID/Nigeria Increasing Access to Education in Northern Nigeria.

[16] Federal Ministry of Education, Nigeria (2006). Implementation guidelines on National school health programme. Federal Ministry of Education. Abuja.

[17] City Population NIGERIA: Administrative Division.

[18] Britannica Federal Capital Territory: administrative territory, Nigeria.

[19] Lwanga SK, Lemeshow S (1991). Sample size determination in health study. World Health Organization Practical Manual, Geneva.

[20] Anderson CL, Cresswell W (1980). School health practice.

[21] Ezeonu CT, Akani NA (2010). Evaluating school health appraisal scheme in primary schools within Abakaliki metropolis, Ebonyi State, Nigeria. Ebonyi Med J.

[22] Mohlabi D, Van Aswegen E, Mokoena J (2010). Barriers to the successful implementation of school health services in the Mpumalanga and Gauteng provinces. South Africa Fam Pract.

[23] Mumah J (2015). Strengthening school health programming in Nairobi City County. A presentation at African Population and Health Research Center roundtable discussion “What works for Girls´ Education”. Nairobi.

[24] Osuorah DIC, Ulasi OT, Ebenebe J, Onah KC, Ndu KI, Ekwochi U, Asinobi NI (2016). The status of school health services?: A comparative study of primary schools in a developing country. Am J Public Heal Res.

[25] Bisi-onyemaechi AI, Akani AN, Ikefuna AN, Tagbo BN, Chinawa JM, Chikani UN (2017). School health services in Enugu East, Nigeria: Perspectives from a resource-poor setting. Healthcare in low-resource settings.

[26] Kuponiyi OT, Amoran OE (2016). School health services and its practice among public and private primary schools in Western Nigeria. BMC Res Notes.

[27] Kansal D, Baliga SS, Mallapur MD, Katti SM (2015). Comparison of school health services among private and government-owned schools of Belgaum. Virtual Health Library.

[28] Kolbe L J (2019). School health as a strategy to improve both public health and education. Annu Rev Public Health.

[29] Adeniran A, Ezeiru S (2016). School health programme practices among private secondary school administrators in an urban Local Government Area in Lagos State, Nigeria. Int J Community MedPublic Heal.

[30] Qureshi FM, Khalid N, Nigah-e-Mumtaz S, Assad T, Noreen K (2018). First aid facilities in the school settings: Are schools able to manage adequately?. Pakistan J Med Sci.

[31] Ofovwe G, Ofili A (2007). Knowledge, attitude and practice of school health programme among headteachers of primary schools in Egor Local Government Area of Edo. Ann Afr Med.

[32] Asiabaka IP (2008). Assessment Of facility needs of government primary schools in Imo State, Nigeria?: Some neglected areas. New York Sci J.

[33] Ojugo A (2005). Status of health appraisal services for primary school children in Edo State, Nigeria. Nig Int Elect J Hlth Edu.

[34] Abodunrin OL, Adeoye OA, Adeomi AA, Osundina FF (2014). Practices, scope and determinants of school health services in Osun State, Nigeria. British Journal of Medicine & Medical Research.

[35] Lund PM (2001). Health and education of children with albinism in Zimbabwe. Health Educ Res.

[36] Munoz-baell IM, Alvarez-dardet C, Ruiz MT, Ferreiro-lago E, Aroca-fernandez EVA (2008). Setting the Stage for School Health-promoting Programmes for Deaf Children in Spain. Health Promot Int.

[37] Mackroth MS, Irwin K, Vandelaer J, Hombach J, Eckert LO (2010). Immunizing School-age Children and Adolescents: Experience from Low and Middle-Income Countries. Vaccine.

[38] Vandelaer J, Olaniran M (2015). Using a School-based Approach to Deliver Immunization — Global Update. Vaccine.

[39] Gothefors L (2008). The Impact of Vaccines in Low-and High-Income Countries. Ann Nestle.

[40] Monney I, Agyei D, Owusu W (2013). Hygienic Practices among Food Vendors in Educational Institutions in Ghana: The Case of Konongo. Foods.

